# Disparities in Preconception Health Indicators —  Behavioral Risk Factor Surveillance System, 2013–2015, and Pregnancy Risk Assessment Monitoring System, 2013–2014

**DOI:** 10.15585/mmwr.ss6701a1

**Published:** 2018-01-19

**Authors:** Cheryl Robbins, Sheree L. Boulet, Isabel Morgan, Denise V. D’Angelo, Lauren B. Zapata, Brian Morrow, Andrea Sharma, Charlan D. Kroelinger

**Affiliations:** 1 Division of Reproductive Health, National Center for Chronic Disease Prevention and Health Promotion, CDC; 2 U.S. Public Health Service Commissioned Corps

## Abstract

**Problem/Condition:**

Preconception health is a broad term that encompasses the overall health of nonpregnant women during their reproductive years (defined here as aged 18*–*44 years). Improvement of both birth outcomes and the woman’s health occurs when preconception health is optimized. Improving preconception health before and between pregnancies is critical for reducing maternal and infant mortality and pregnancy-related complications. The National Preconception Health and Health Care Initiative’s Surveillance and Research work group suggests ten prioritized indicators that states can use to monitor programs or activities for improving the preconception health status of women of reproductive age. This report includes overall and stratified estimates for nine of these preconception health indicators.

**Reporting Period:**

2013*–*2015.

**Description of Systems:**

Survey data from two surveillance systems are included in this report. The Behavioral Risk Factor Surveillance System (BRFSS) is an ongoing state-based, landline and cellular telephone survey of noninstitutionalized adults in the United States aged ≥18 years that is conducted by state and territorial health departments. BRFSS is the main source of self-reported data for states on health risk behaviors, chronic health conditions, and preventive health services primarily related to chronic disease in the United States. The Pregnancy Risk Assessment Monitoring System (PRAMS) is an ongoing U.S. state- and population-based surveillance system administered collaboratively by CDC and state health departments. PRAMS is designed to monitor selected maternal behaviors, conditions, and experiences that occur before, during, and shortly after pregnancy that are self-reported by women who recently delivered a live-born infant.

This report summarizes BRFSS and PRAMS data on nine of 10 prioritized preconception health indicators (i.e., depression, diabetes, hypertension, current cigarette smoking, normal weight, recommended physical activity, recent unwanted pregnancy, prepregnancy multivitamin use, and postpartum use of a most or moderately effective contraceptive method) for which the most recent data are available. BRFSS data from all 50 states and the District of Columbia were used for six preconception health indicators: depression, diabetes (excluded if occurring only during pregnancy or if limited to borderline/prediabetes conditions), hypertension (excluded if occurring only during pregnancy or if limited to borderline/prehypertension conditions), current cigarette smoking, normal weight, and recommended physical activity. PRAMS data from 30 states, the District of Columbia, and New York City were used for three preconception health indicators: recent unwanted pregnancy, prepregnancy multivitamin use, and postpartum use of a most or moderately effective contraceptive method by women or their husbands or partners (i.e., male or female sterilization, hormonal implant, intrauterine device, injectable contraceptive, oral contraceptive, hormonal patch, or vaginal ring). Heavy alcohol use during the 3 months before pregnancy also was included in the prioritized set of 10 indicators, but PRAMS data for each reporting area are not available until 2016 for that indicator. Therefore, estimates for heavy alcohol use are not included in this report. All BRFSS preconception health estimates are based on 2014*–*2015 data except two (hypertension and recommended physical activity are based on 2013 and 2015 data). All PRAMS preconception health estimates rely on 2013*–*2014 data. Prevalence estimates of indicators are reported for women aged 18*–*44 years overall, by age group, race-ethnicity, health insurance status, and reporting area. Chi-square tests were conducted to assess differences in indicators by age group, race/ethnicity, and insurance status.

**Results:**

During 2013*–*2015, prevalence estimates of indicators representing risk factors were generally highest and prevalence estimates of health-promoting indicators were generally lowest among older women (35*–*44 years), non-Hispanic black women, uninsured women, and those residing in southern states. For example, prevalence of ever having been told by a health care provider that they had a depressive disorder was highest among women aged 35*–*44 years (23.1%) and lowest among women aged 18*–*24 years (19.2%). Prevalence of postpartum use of a most or moderately effective method of contraception was lowest among women aged 35*–*44 years (50.6%) and highest among younger women aged 18*–*24 years (64.9%). Self-reported prepregnancy multivitamin use and getting recommended levels of physical activity were lowest among non-Hispanic black women (21.6% and 42.8%, respectively) and highest among non-Hispanic white women (37.8% and 53.8%, respectively). Recent unwanted pregnancy was lowest among non-Hispanic white women and highest among non-Hispanic black women (5.0% and 11.6%, respectively). All but three indicators (diabetes, hypertension, and use of a most or moderately effective contraceptive method) varied by insurance status; for instance, prevalence of current cigarette smoking was higher among uninsured women (21.0%) compared with insured women (16.1%), and prevalence of normal weight was lower among women who were uninsured (38.6%), compared with women who were insured (46.1%). By reporting area, the range of women reporting ever having been told by a health care provider that they had diabetes was 5.0% (Alabama) to 1.9% (Utah), and women reporting ever having been told by a health care provider that they had hypertension ranged from 19.2% (Mississippi) to 7.0% (Minnesota).

**Interpretation:**

Preconception health risk factors and health-promoting indicators varied by age group, race/ethnicity, insurance status, and reporting area. These disparities highlight subpopulations that might benefit most from interventions that improve preconception health.

**Public Health Action:**

Eliminating disparities in preconception health can potentially reduce disparities in two of the leading causes of death in early and middle adulthood (i.e., heart disease and diabetes). Public health officials can use this information to provide a baseline against which to evaluate state efforts to improve preconception health.

## Introduction

Preconception health is a broad term that encompasses the overall health of nonpregnant women during their reproductive years (defined here as aged 18*–*44 years). Improvement of both birth outcomes and the woman’s health occurs when preconception health is optimized. Improving preconception health before and between pregnancies is critical for reducing maternal and infant mortality and pregnancy-related complications (*1*). Eliminating racial disparities in preconception health can potentially reduce disparities in two of the leading causes of death in early and middle adulthood (i.e., heart disease and diabetes) (*2*). Surveillance and epidemiologic research on women of reproductive age suggests that two thirds are presumed to be fecund (i.e., can become pregnant) (*3*) and that approximately half of their pregnancies are unintended (*4*) and are affected by chronic health and behavioral risk factors that are associated with adverse maternal and infant outcomes (*5*). Uncontrolled chronic conditions (e.g., hypertension and diabetes) are associated with severe maternal and infant morbidity and mortality (*6*–*9*). Smoking, alcohol consumption, and inadequate folic acid intake are associated with preterm birth, low birthweight, fetal growth restriction, fetal alcohol spectrum disorders, neural tube defects, other birth defects, and even infant death (*10*–*14*). Moreover, evidence-based interventions for these risk factors can improve the health outcomes of women and infants (*14*–*19*).

CDC and other federal agencies, professional health and clinical organizations, and national experts recognize the importance of promoting and monitoring the preconception health status of women of reproductive age in the United States through formal recommendations (*6*,*20*–*23*). In 2007, the first state-based surveillance summary of preconception health status used 2004 Pregnancy Risk Assessment Monitoring System (PRAMS) data (*24*). After that, state-level policy and program leaders and epidemiologists identified 45 core state preconception health indicators (*25*). A second state-based surveillance summary used 2009 PRAMS and Behavioral Risk Factor Surveillance System (BRFSS) data and included estimates for 39 of the 45 core preconception health indicators (*26*).

In 2016, to fill the need for more focused measurement of preconception health, the National Preconception Health and Health Care Initiative’s Surveillance and Research work group prioritized available measures of preconception health indicators and suggested 10 indicators for states to consider monitoring (*27*). These 10 indicators are depression, diabetes, hypertension, current cigarette smoking, normal weight, recommended physical activity, recent unwanted pregnancy, prepregnancy multivitamin use, postpartum use of a most or moderately effective contraceptive method, and prepregnancy heavy alcohol use. The prioritization excluded measures of demographic indicators (e.g., health insurance coverage) because these and other measures of social determinants of health represent important stratification variables that can be used to identify opportunities to improve equity and reduce disparities in preconception health. Adverse pregnancy outcomes vary by age, race/ethnicity, and insurance status (*28*–*30*). Therefore, in support of ongoing efforts to monitor preconception health, this report includes overall and stratified estimates of nine of the 10 prioritized preconception health indicators using the most recent available PRAMS and BRFSS data. The purpose of this analysis is to provide baseline estimates that can serve as benchmarks for monitoring the preconception health status of women of reproductive age. The data also can be used by states for needs assessments, program planning, and evaluation.

## Methods

### Description of the Surveillance Systems

#### BRFSS Description and Data Collection

BRFSS is an ongoing state-based survey of noninstitutionalized adults in the United States aged ≥18 years that has been conducted since 1984 by state and territorial health departments with assistance from CDC. This report examines BRFSS data from all 50 states and the District of Columbia. BRFSS aims to conduct 4,000 interviews per reporting area each year and is the main source of state-specific data on health risk behaviors, chronic health conditions, and use of preventive health services primarily related to chronic disease and injury in the United States. Details on BRFSS methods are available on the BRFSS website (http://www.cdc.gov/brfss).

BRFSS uses a multistage sampling design created on the basis of random-digit–dialing methods to select representative samples in each reporting area. Trained interviewers administer the BRFSS questionnaire using a computer-assisted telephone interviewing system. This report uses only BRFSS data from the core component (i.e., the annual standard core and a biannual rotating core) of the questionnaire, which contains questions that are asked in all participating jurisdictions.

This report includes data from nonpregnant women aged 18*–*44 years residing in 51 reporting areas (the 50 U.S. states and the District of Columbia) during 2013*–*2015 (the most recent data available). For indicators relying on annual standard core questions (i.e., questions that are used every year), estimates were based on responses from 118,219 women during 2014*–*2015. For indicators that were based on the biannual rotating core survey, data from 2013 and 2015 were combined and were based on responses from 127,592 women. The median response rates (percentage eligible for whom an interview was completed) across reporting areas for landlines and cell phones, respectively, were 49.6% and 37.8% (2013), 48.7% and 40.5% (2014), and 48.2% and 47.2% (2015).

#### PRAMS Description and Data Collection

PRAMS is an ongoing U.S. state- and population-based surveillance system that was established in 1987. PRAMS was designed to monitor selected self-reported maternal behaviors, conditions, and experiences that occur before, during, and shortly after pregnancy among women who deliver live-born infants in participating U.S. states, New York City, and the District of Columbia. PRAMS is administered by CDC in collaboration with state health departments. Details on the PRAMS methods are available on the PRAMS website (http://www.cdc.gov/PRAMS).

All PRAMS reporting areas use a standardized data-collection method. PRAMS reporting areas select a monthly sample of 100–300 new mothers from recent birth certificates. Annual sample sizes in 2013*–*2014 (the most recent data available) ranged from 1,000 to 4,000 per reporting area. PRAMS uses mixed-mode data collection including self-administered, mailed questionnaires and follow-up phone calls. The first questionnaire is usually mailed 2 to 3 months after the delivery of a live-born infant to allow for collection of information about postpartum maternal and infant experiences. Survey data are linked to selected birth certificate data and weighted for sample design, nonresponse, and noncoverage. All PRAMS indicators in this report are based on core questions, which are used by all reporting areas.

All participating PRAMS reporting areas that had achieved an overall weighted response rate of ≥60% were included. The response rate was calculated as the number of responding mothers divided by the number of mothers sampled, adjusted by the sample design. This report includes PRAMS data from 59,095 women residing in 30 states, the District of Columbia, and New York City, representing approximately 55% of 8 million live births in the United States during 2013*–*2014.

### Selection of Indicators

This report includes data on the prioritized set of preconception health indicators that were selected based on 1) prevalence, 2) relation to professional recommendations, Healthy People 2020 objectives, or CDC Winnable Battles, 3) measurement simplicity, 4) data completeness, and 5) stakeholder input from maternal and child-health directors and epidemiologists working in state health departments (*27*). The 10 indicators include depression (BRFSS), diabetes (excluded if occurring only during pregnancy or borderline/prediabetes) (BRFSS), hypertension (excluded if occurring only during pregnancy or borderline/prehypertension) (BRFSS), current cigarette smoking (BRFSS), normal weight (BRFSS), recommended physical activity (BRFSS), recent unwanted pregnancy (PRAMS), prepregnancy multivitamin use (PRAMS), postpartum use of a most or moderately effective contraceptive method (PRAMS), and heavy alcohol consumption (PRAMS). Some indicators represent risk factors that are associated with maternal morbidity and mortality and with adverse infant outcomes, and others are health-promoting indicators that foster positive pregnancy and infant outcomes and long-term health. Estimates for the six BRFSS indicators include data from 51 reporting areas, and estimates for the four PRAMS indicators include data from 32 reporting areas, with the exception of heavy alcohol use. Estimates of heavy alcohol use are not included in this report of 2013*–*2014 data because the Phase 7 PRAMS (2012*–*2015) response options only permitted estimates of seven or more drinks per week, and the prioritized alcohol use indicator is defined as eight or more drinks per week, which is a better measure of heavy alcohol use (*31*). PRAMS data that will allow for calculation of eight or more drinks per week will be available on the core questionnaire beginning with 2016 data.

### Data Analysis

All BRFSS indicators were estimated for nonpregnant women of reproductive age (18–44 years), and PRAMS indicators were estimated for postpartum women with recent live births. For each reporting area, weighted prevalence estimates and 95% confidence intervals (CIs) of indicators are presented overall and stratified by age group (18–24, 25–34, and 35–44 years) and race/ethnicity (non-Hispanic white, non-Hispanic black, non-Hispanic other, and Hispanic). Estimates also were stratified by health insurance status (yes or no), which was captured differently between the two data sources. The core BRFSS survey asks respondents whether they have any kind of health care coverage, including health insurance, prepaid plans such as health maintenance organizations, or government plans such as Medicare or Indian Health Service (IHS). Respondents who answered “yes” were defined as having health insurance. The core PRAMS questionnaire asks respondents what kind of health insurance they had during the month before they got pregnant. Response options coded as “yes” included private, Medicaid, other government plans such as TRICARE, military health care, IHS or tribal, and other kinds of health insurance. The response “I did not have any health insurance during the month before I got pregnant” was coded as “no.”

The overall median, minimum, and maximum values for all reporting areas also were calculated and are available at https://stacks.cdc.gov/view/cdc/cdc:49859. Chi-square tests were conducted to assess overall differences in indicators by age group, race/ethnicity, and insurance status. Differences were considered statistically significant when the p value was <0.05. Tests of statistical significance were not conducted by reporting area, but reporting areas with the highest and lowest estimates were noted. In accordance with the reporting policy of the specific surveillance system, when the denominator was <30 respondents for PRAMS or <50 respondents for BRFSS, prevalence estimates were not reported (Supplementary Tables S1–S9, https://stacks.cdc.gov/view/cdc/cdc:49859). In addition, prevalence estimates made on the basis of 30–59 respondents (PRAMS) or 50–59 respondents (BRFSS) are footnoted because they might not be reliable, and all missing observations are excluded. To account for the complex survey designs, all analyses were conducted using statistical software. BRFSS analyses were conducted using weights and strata with SAS-Callable SUDAAN release 11.0 (Research Triangle Institute, Cary, North Carolina). PRAMS analyses were conducted using weighted data with Stata release 14.0 (StataCorp LP, College Station, Texas).

## Results

### Depression (BRFSS)

Depression was self-reported by women and defined as ever being told by a health care provider that they had a depressive disorder. During 2014*–*2015, among nonpregnant women, the overall prevalence of depression was 21.9% (Table 1). For all reporting areas combined, the prevalence of this indicator varied significantly by age group, race/ethnicity, and insurance status. Prevalence by age group ranged from 19.2% in the youngest age group (18*–*24 years) to 23.1% in the oldest age group (35*–*44 years). Prevalence of depression was 14.8% among women who identified themselves as other race/ethnicity, 15.5% among Hispanic women, 16.2% among non-Hispanic black women, and 27.0% among non-Hispanic white women. Prevalence of reported depression was lower among uninsured women (20.3%) compared with insured women (22.3%). By reporting area, the lowest prevalence estimate was 13.6% (Hawaii), and the highest estimate was 35.1% (Oregon); the median was 25.9% (Supplementary Table S1, https://stacks.cdc.gov/view/cdc/cdc:49859).

**TABLE 1:** Prevalence of preconception health indicators among nonpregnant reproductive-aged women (18–44 years), by age group, race/ethnicity, and insurance — Behavioral Risk Factor Surveillance System, United States, 2013–2015*

Characteristic	Depression^†^ (2014–2015)	Diabetes^†§^ (2014–2015)	Hypertension^†§¶^ (2013, 2015)	Current cigarette smoking** (2014–2015)	Normal weight^††^ (2014–2015)	Recommended physical activity^¶§§^ (2013, 2015)
	**% (95% CI)**	**% (95% CI)**	**% (95% CI)**	**% (95% CI)**	**% (95% CI)**	**% (95% CI)**
**Age group (yrs) ^¶¶^**						
18–24	19.2 (18.4–20.1)	1.0 (0.8–1.2)	5.0 (4.5–5.4)	13.4 (12.7–14.1)	57.0 (55.9–58.2)	53.3 (52.1–54.4)
25–34	22.6 (22.0–23.3)	2.4 (2.1–2.7)	9.2 (8.7–9.7)	19.5 (18.9–20.1)	42.7 (41.8–43.5)	49.7 (48.9–50.6)
35–44	23.1 (22.5–23.7)	5.3 (4.9–5.6)	17.0 (16.4–17.6)	16.8 (16.3–17.4)	37.9 (37.2–38.7)	49.0 (48.2–49.8)
**Race/Ethnicity^¶¶^**						
White	27.0 (26.5–27.6)	2.6 (2.4–2.8)	10.2 (9.8–10.5)	21.1 (20.6–21.6)	49.0 (48.3–49.6)	53.8 (53.2–54.4)
Black	16.2 (15.1–17.2)	4.5 (4.0–5.1)	18.3 (17.3–19.3)	15.6 (14.5–16.7)	30.0 (28.6–31.5)	42.8 (41.3–44.3)
Hispanic	15.5 (14.6–16.4)	3.6 (3.2–4.1)	9.5 (8.7–10.3)	8.9 (8.2–9.6)	37.2 (35.9–38.6)	46.0 (44.6–47.4)
Other	14.8 (13.6–16.1)	2.4 (1.9–2.8)	8.0 (7.1–9.0)	11.3 (10.3–12.4)	57.6 (55.6–59.6)	50.3 (48.2–52.4)
Insurance^***†††^						
Yes	22.3 (21.8–22.7)	3.1 (2.9–3.2)	10.8 (10.5–11.2)	16.1 (15.7–16.5)	46.1 (45.6–46.7)	51.8 (51.2–52.4)
No	20.3 (19.2–21.3)	3.2 (2.8–3.6)	11.5 (10.8–12.2)	21.0 (20.0–22.0)	38.6 (37.2–40.0)	44.0 (42.7–45.3)
**Overall**	**21.9 (21.5–22.3)**	**3.1 (2.9–3.2)**	**10.9 (10.6–11.2)**	**16.9 (16.5–17.2)**	**44.9 (44.4–45.5)**	**50.4 (49.9–50.9)**

**Abbreviation:** CI = confidence interval.

* For indicators relying on annual standard core questions (i.e., questions that are asked annually by all states), estimates are based on 2014–2015 data. For indicators that are based on the biannual rotating core survey, CDC combined years 2013 and 2015; includes 50 U.S. states and the District of Columbia. Data self-reported by women aged 18–44 years.

^†^ Self-report of ever having been told by a health care provider that they have the condition.

^§^ Excluded if occurring only during pregnancy.

^¶^ Hypertension and physical activity questions are included as part of the biannual rotating core that is administered in odd years; therefore, 2013 and 2015 data were used.

** Defined as smoking 100 or more cigarettes in a lifetime and currently smoking cigarettes every day or some days at the time of the interview.

^††^ Normal weight was defined as having a body mass index of 18.5–24.9 kg/m^2^ as determined by self-reported weight and height.

^§§^ Participation in enough moderate and/or vigorous physical activity in a usual week was defined as meeting the U.S. Department of Health and Human Services recommended levels of aerobic physical activity. Respondents were classified as meeting recommendations if they reported at least 150 minutes per week of moderate-intensity activity, or at least 75 minutes per week of vigorous-intensity activity, or a combination of moderate-intensity and vigorous-intensity activity (where vigorous activity minutes are multiplied by two) totaling at least 150 minutes per week.

^¶¶^ In Chi-square tests, differences by age and by race/ethnicity are significant at p<0.05 for all indicators.

*** Defined as having any kind of health care coverage, including prepaid plans such as health maintenance organizations or government plans such as Medicare or Indian Health Service.

^†††^ In Chi-square tests, differences by insurance are significant at p<0.05 for all indicators except diabetes and hypertension.

### Diabetes (BRFSS)

Diabetes was self-reported by women and defined as ever being told by a health care provider that they had diabetes (excluded if occurring only during pregnancy or borderline/prediabetes). During 2014*–*2015, among nonpregnant women, the estimated overall prevalence of diabetes was 3.1% (Table 1). For all reporting areas combined, the prevalence of this indicator varied significantly by age group and race/ethnicity, but not by insurance status. Prevalence by age group ranged from 1.0% in the youngest age group (18*–*24 years) to 5.3% in the oldest age group (35*–*44 years). Prevalence of diabetes was 2.4% among women who identified themselves as other race/ethnicity, 2.6% among non-Hispanic white women, 3.6% among Hispanic women, and 4.5% among non-Hispanic black women. By reporting area, the lowest prevalence estimate was 1.9% (Utah), and the highest estimate was 5.0% (Alabama); the median was 3.1% (Supplementary Table S2, https://stacks.cdc.gov/view/cdc/cdc:49860).

### Hypertension (BRFSS)

Hypertension was self-reported by women and defined as ever being told by a health care provider that they had hypertension (excluded if occurring only during pregnancy or borderline/prehypertension). During 2013 and 2015, among nonpregnant women, the estimated overall prevalence of hypertension was 10.9% (Table 1). For all reporting areas combined, the prevalence of this indicator varied significantly by age group and race/ethnicity, but not by insurance status. Prevalence by age group ranged from 5.0% among the youngest age group (18*–*24 years) to 17.0% among the oldest age group (35*–*44 years). Prevalence of hypertension was 8.0% among women who identified themselves as other race/ethnicity, 9.5% among Hispanic women, 10.2% among non-Hispanic white women, and 18.3% among non-Hispanic black women. By reporting area, the lowest prevalence estimate was 7.0% (Minnesota), and the highest estimate was 19.2% (Mississippi); the median was 9.9% (Supplementary Table S3, https://stacks.cdc.gov/view/cdc/cdc:49861).

### Current Cigarette Smoking (BRFSS)

Current cigarette smoking was self-reported by women and defined as current cigarette smoking (at the time of the survey) every day or some days among women who had ever smoked 100 or more cigarettes in their lifetime. During 2014*–*2015, among nonpregnant women aged 18*–*44 years, the estimated overall prevalence of current cigarette smoking was 16.9% (Table 1). For all reporting areas combined, the prevalence of this indicator varied significantly by age group, race/ethnicity, and insurance status. Prevalence of current cigarette smoking by age group ranged from 13.4% (18*–*24 years) to 19.5% (25*–*34 years). Prevalence of current cigarette smoking was lowest among Hispanic women (8.9%), followed by women who identified themselves as other race/ethnicity (11.3%), non-Hispanic black women (15.6%), and highest among non-Hispanic white women (21.1%). Current cigarette smoking was less prevalent among insured women (16.1%) compared with uninsured women (21.0%). By reporting area, the lowest prevalence estimate was 8.4% (California), and the highest estimate was 33.7% (West Virginia); the median was 18.8% (Supplementary Table S4, https://stacks.cdc.gov/view/cdc/cdc:49862).

### Normal Weight (BRFSS)

Normal weight was defined as having a body mass index of 18.5*–*24.9 kg/m^2^ (as determined by self-reported weight and height). During 2014*–*2015, among nonpregnant women aged 18*–*44 years, the estimated overall prevalence of normal weight was 44.9% (Table 1). For all reporting areas combined, the prevalence of this indicator varied significantly by age group, race/ethnicity, and insurance status. Prevalence of normal weight by age group was 57.0% (18*–*24 years), 42.7% (25*–*34 years), and 37.9% (35*–*44 years). Prevalence of normal weight was 57.6% among women who identified themselves as other race/ethnicity, 49.0% among non-Hispanic white women, 37.2% among Hispanic women, and 30.0% among non-Hispanic black women. Normal weight was more prevalent among insured women (46.1%) than uninsured women (38.6%). By reporting area, the lowest prevalence estimate was 33.0% (Mississippi), and the highest estimate was 53.9% (Massachusetts); the median was 44.2% (Supplementary Table S5, https://stacks.cdc.gov/view/cdc/cdc:49863).

### Recommended Physical Activity (BRFSS)

Participating in recommended levels of physical activity each week was defined as getting at least 150 minutes per week of moderate-intensity activity, at least 75 minutes per week of vigorous-intensity activity, or a combination of moderate-intensity and vigorous-intensity activity (where vigorous activity minutes are multiplied by two) totaling at least 150 minutes per week, according to self report (*32*). During 2013 and 2015, among nonpregnant women, the estimated overall prevalence of participating in the recommended level of physical activity was 50.4% (Table 1). For all reporting areas combined, the prevalence of this indicator varied significantly by age group, race/ethnicity, and insurance status. Prevalence of recommended physical activity by age group ranged from 53.3% (18*–*24 years) to 49.7% (25–34 years) and 49.0% (35*–*44 years). Prevalence of recommended physical activity was 53.8% among non-Hispanic white women, 50.3% among women who identified themselves as other race/ethnicity, 46.0% among Hispanic women, and 42.8% among non-Hispanic black women. Meeting physical activity recommendations was more prevalent among insured women (51.8%) than uninsured women (44.0%). By reporting area, prevalence estimates ranged from a low of 39.6% (Mississippi) to 61.8% (Oregon); the median was 51.5% (Supplementary Table S6, https://stacks.cdc.gov/view/cdc/cdc:49864).

### Recent Unwanted Pregnancy (PRAMS)

Recent unwanted pregnancy was defined as a pregnancy among women who reported that just before they got pregnant with their most recent live-born infant, they did not want to be pregnant then or at any time in the future. During 2013–2014, among women aged 18*–*44 years with a recent live birth, the estimated overall prevalence of women who reported that the pregnancy was unwanted was 6.1% (Table 2). For all reporting areas combined, the prevalence of this indicator varied significantly by age group, race/ethnicity, and insurance status. Specifically, the lowest prevalence of recent unwanted pregnancy was reported by women aged 25*–*34 years (4.9%), compared with 6.4% among those aged 18*–*24 years and 9.8% among those aged 35*–*44 years. In addition, 5.0% of non-Hispanic white women reported that the pregnancy was unwanted, compared with 6.0% of women who identified themselves as other race/ethnicity, 6.4% of Hispanic women, and 11.6% of non-Hispanic black women. Recent unwanted pregnancy was less prevalent among insured women (5.8%) compared with uninsured women (7.3%). Prevalence estimates ranged from a low of 3.4% (Washington) to a high of 10.9% (Arkansas); the median was 6.0% (Supplementary Table S7, https://stacks.cdc.gov/view/cdc/cdc:49865).

**TABLE 2:** Prevalence of preconception health indicators among reproductive-aged women (aged 18–44 years) with a recent live birth, by age group, race/ethnicity, and insurance — Pregnancy Risk Assessment Monitoring System, United States, 2013 and 2014*

Characteristic	Recent unwanted pregnancy^†^	Prepregnancy multivitamin use^§^	Postpartum use of effective contraception^¶^
	**% (95% CI)**	**% (95% CI)**	**% (95% CI)**
Age group (yrs)**			
18–24	6.4 (5.8–7.1)	17.9 (17.0–18.9)	64.9 (63.6–66.2)
25–34	4.9 (4.6–5.3)	37.4 (36.6–38.2)	55.1 (54.3–55.9)
35–44	9.8 (8.9–10.8)	45.4 (43.8–46.9)	50.6 (49.0–52.3)
Race/Ethnicity**			
White	5.0 (4.6–5.4)	37.8 (37.1–38.6)	56.8 (55.9–57.6)
Black	11.6 (10.4–12.8)	21.6 (20.2–23.2)	64.9 (63.1–66.7)
Hispanic	6.4 (5.6–7.3)	26.2 (24.8–27.7)	59.3 (57.5–61.0)
Other	6.0 (5.2–6.8)	31.7 (30.1–33.4)	44.6 (42.8–46.5)
**Prepregnancy insurance^††§§^**			
Yes	5.8 (5.5–6.1)	37.4 (36.7–38.1)	56.7 (56.0–57.4)
No	7.3 (6.6–8.1)	17.1 (16.0–18.2)	57.9 (56.4–59.5)
**Overall**	**6.1 (5.8–6.4)**	**33.6 (33.0–34.2)**	**56.9 (56.3–57.6)**

**Abbreviation:** CI = 95% confidence interval.

* Includes Alabama, Alaska, Arkansas, Colorado, Delaware, Georgia, Hawaii, Illinois, Iowa, Maine, Maryland, Massachusetts, Michigan, Minnesota, Missouri, Nebraska, New Hampshire, New Jersey, New Mexico, New York, New York City, Oklahoma, Oregon, Pennsylvania, Rhode Island, Tennessee, Utah, Vermont, Washington, West Virginia, Wisconsin, and Wyoming. Data self-reported by women aged 18–44 years who recently had a live birth.

^†^ Defined as a pregnancy among women who reported that just before they got pregnant with their most recent live-born infant, they did not want to be pregnant then or at any time in the future.

^§^ Defined as taking a multivitamin, prenatal vitamin, or folic acid supplement every day of the month before pregnancy.

^¶^ Includes male or female sterilization, implant, intrauterine device, injectable, pill, patch, or ring.

** In Chi-square tests, differences by age and by race/ethnicity are significant at p<0.05 for all indicators.

^††^ Defined as having private, Medicaid, other government plans such as TRICARE, military health care, IHS or tribal, and other kinds of health insurance during the month before pregnancy.

^§§^ In Chi-square tests, differences by insurance are significant at p<0.05 for all indicators except postpartum use of effective contraception.

### Prepregnancy Multivitamin Use (PRAMS)

Multivitamin use during the month before pregnancy was defined as taking a multivitamin, prenatal vitamin, or folic acid supplement every day of the month before pregnancy. During 2013*–*2014, among women aged 18*–*44 years with a recent live birth, the estimated overall prevalence of women who reported multivitamin use during the month before pregnancy was 33.6% (Table 2). For all reporting areas combined, the prevalence of this indicator varied significantly by age group, race/ethnicity, and insurance status. Specifically, prevalence of prepregnancy multivitamin use was highest among women aged 35*–*44 years (45.4%), followed by women aged 25*–*34 years (37.4%), and lowest among women aged 18*–*24 years (17.9%). In addition, prepregnancy multivitamin use was highest among non-Hispanic white women (37.8%) and women who identified themselves as other race/ethnicity (31.7%) and lowest among Hispanic women (26.2%) and non-Hispanic black women (21.6%). Multivitamin use during the month before pregnancy was more prevalent among insured women (37.4%) than uninsured women (17.1%). By reporting area, the lowest prevalence estimate was 23.3% (Georgia), and the highest estimate was 41.2% (Massachusetts); the median was 34.3% (Supplementary Table S8, https://stacks.cdc.gov/view/cdc/cdc:49866).

### Postpartum Use of Contraception (PRAMS)

Postpartum use of a most or moderately effective method of contraception was defined as current use of one or more of the following birth control methods by women or their husbands or partners: male or female sterilization, implant, intrauterine device, injectable, pill, patch, or vaginal ring. The most effective methods (i.e., male or female sterilization, implant, and intrauterine device) have a failure rate that is <1% with typical use, and moderately effective methods (shot, pill, patch, ring or diaphragm) include those with typical failure rates of 18%–28%. Diaphragm was not a response option in these data (*33*). During 2013*–*2014, among women aged 18*–*44 years with a recent live birth, the estimated overall prevalence of self-reported use of a most or moderately effective contraceptive method was 56.9% (Table 2). For all reporting areas combined, the prevalence of this indicator varied significantly by age group and race/ethnicity, but not by insurance status. Specifically, 64.9% of women aged 18*–*24 years reported using a most or moderately effective contraceptive method, compared with 55.1% of those aged 25*–*34 years and 50.6% of those aged 35*–*44 years. In addition, 64.9% of non-Hispanic black women reported using a most or moderately effective contraceptive method, compared with 59.3% of Hispanic women, 56.8% of non-Hispanic white women, and 44.6% of women who identified themselves as other race/ethnicity. Prevalence estimates ranged from a low of 40.2% (New York City) to a high of 72.4% (Georgia); the median was 59.1% (Supplementary Table S9, https://stacks.cdc.gov/view/cdc/cdc:49867).

## Discussion

This report uses 2013–2014 PRAMS and 2013–2015 BRFSS data (the most recent available) to provide estimates for nine of 10 indicators that were recently identified as priority measures for monitoring preconception health among women of reproductive age (*27*). Patterns of disparities emerged by age group, race/ethnicity, insurance status, and reporting area. Specifically, risk factors were generally more prevalent and health-promoting indicators less prevalent among older women, non-Hispanic black women, and women who were uninsured. In addition, the highest prevalence of risk factor indicators and the lowest prevalence of health promoting indicators were noted in southern states (Alabama, Arkansas, Georgia, Mississippi, and West Virginia) for seven of nine of the preconception health indicators (*34*). Taken together, these indicators suggest that many women could benefit from interventions to optimize health.

These disparities by age, race/ethnicity, insurance, and reporting area are consistent with previously published reports. Other epidemiologic reports have highlighted higher prevalence of chronic conditions (e.g., diabetes and hypertension) among older women (*24*,*26*). Consistent with previous findings, this report described larger proportions of older women reporting multivitamin use before pregnancy compared with younger women (*24*,*26*). Similar to previous surveillance summaries, this report found that non-Hispanic black women reported more diagnoses of diabetes and hypertension, less recommended physical activity or multivitamin use before pregnancy, and lower prevalence of normal weight (*24*,*26*). Only one surveillance summary has previously reported estimates for preconception health indicators by insurance status (*24*). That report used PRAMS data and included estimates of prepregnancy diabetes, hypertension, obesity, current use of any tobacco, multivitamins, and postpartum depression and contraception stratified by prepregnancy insurance type (private, Medicaid, or none). Although measurement of the indicators in that report differed from measurement in the current report, the 2004 data also showed that women with no insurance had higher prevalence of many risk factors compared with those who had either Medicaid or private insurance (*24*). In addition, PRAMS and BRFSS preconception health indicators from 2008–2009 varied by geography, with the highest prevalence of health problems and risky behaviors generally occurring in the southern states (*26*). For example, in both the current report and the report with 2008–2009 data, the highest prevalence of current cigarette smoking by geographic area was reported in West Virginia.

Health inequity, defined as “a subset of health inequalities that are modifiable and associated with social disadvantage, and considered ethically unfair” (*35*), was evidenced by the disparities across indicators for non-Hispanic black women. For example, the findings that non-Hispanic black women had the highest prevalence of diabetes and hypertension and lowest prevalence of normal weight suggest a possible syndemic, defined as “two or more disease states that adversely interact with each other, negatively affecting the mutual course of each disease trajectory, enhancing vulnerability, and which are made more deleterious by experienced inequities” (*36*).

Addressing preconception health indicators might require system changes in clinical care settings and in public health. Transformative, system-level changes require partnerships for implementing interventions and programs, and consistent, reliable data for benchmarking and monitoring progress (*37*). For example, establishing stakeholder partnerships among health departments, insurers, and clinicians is a key strategy for all 27 states and territories participating in the Association of State and Territorial Health Officials’ Learning Community and facilitates critical activities such as data-sharing, policy development, and workforce training (*38*). Because of the known associations between the preconception health indicators and maternal and infant morbidity and mortality (*6*–*9*), uniform preconception health surveillance efforts across states can facilitate efforts to improve maternal health and birth outcomes.

## Limitations

The findings in this report are subject to at least four limitations. First, all self-reported data are subject to recall and social desirability bias. Second, this report included estimates of several chronic conditions (i.e., depression, diabetes, and hypertension), which might be undiagnosed because diagnosis is contingent on use of health care. Therefore, proportions of women who reported having those conditions in this surveillance summary are likely underestimated. Third, these results are not generalizable to all women. PRAMS data were not available for all states. In addition, PRAMS surveys women who delivered live births and does not represent women who had miscarriages or stillbirths, who might have poorer preconception health. The BRFSS response rate varies by state, and no minimum threshold was required for inclusion. Therefore, BRFSS estimates in states with low response rates might not be representative of all women aged 18–44 years in those states. Finally, use of a dichotomous measure of insurance might have masked potential differences by public versus private insurance.

## Conclusion

The condensed, prioritized set of preconception health indicators might increase the use and impact of preconception health surveillance over time. Collectively, the preconception health indicators provide a snapshot of the health status among U.S. women aged 18*–*44, which can be used as a baseline against which to evaluate state efforts to improve health in these women. These results suggest the need to enhance existing collaborations and identify new opportunities for addressing the health needs of women of reproductive age to improve racial and geographic disparities.
