# *Erratum*: Vol. 67, No. SS-1

**DOI:** 10.15585/mmwr.mm6716a6

**Published:** 2018-04-27

In the Surveillance Summary “Disparities in Preconception Health Indicators — Behavioral Risk Factor Surveillance System, 2013*–*2015, and Pregnancy Risk Assessment Monitoring System, 2013*–*2014,” on page 7, the second sentence under the heading “Postpartum Use of Contraception (PRAMS)” should have read “The most effective methods (i.e., male or female sterilization, implant, and intrauterine device) have a failure rate that is <1% with typical use, and moderately effective methods (shot, pill, patch, ring or diaphragm) include those with typical failure rates of **6%***–***12%**.”

